# Novel invasion indices quantify the feed-forward facilitation of tumor invasion by macrophages

**DOI:** 10.1038/s41598-020-57517-6

**Published:** 2020-01-20

**Authors:** Gippeum J. Lim, Suk-Jo Kang, Ji Youn Lee

**Affiliations:** 1 Department of Biological Sciences, Korea Advanced Institute of Science and Technology, 291 Daehak-ro, Yuseong-gu, Daejeon 34141 Republic of Korea; 2 Center for Bioanalysis, Division of Chemical and Medical Metrology, Korea Research Institute of Standards and Science, 267 Gajeong-ro, Yuseong-gu, Daejeon 34113 Republic of Korea

**Keywords:** Cytological techniques, Cancer microenvironment

## Abstract

Quantitative and reliable measurement of cellular invasion is important to understand a range of biological processes such as cancer metastasis and angiogenesis. Spheroid invasion assays are an attractive *in vitro* platform because they effectively mimic the tumor cell invasion of solid tissues. Here, we developed an image analysis–based method to quantify the invasiveness of HT1080 human fibrosarcoma tumor cell spheroids. We segmented a cell-covered area into three subareas using objectively set threshold pixel intensities and calculated invasion indices using these subareas. Comparison with conventional parameters for spheroid invasion assays, such as area, length, and detached cells, showed that our indices present the invasion event at an early time and without being convoluted by proliferation. As an application, we then examined paracrine interactions between LLC1 mouse lung carcinoma cells and Raw264.7 mouse macrophage cells with our developed analysis method. We found that the invasion of tumor spheroids was increased by a macrophage-conditioned medium, concomitantly with a decrease in tumor cell proliferation. Importantly, invasion was further enhanced by a conditioned medium from activated macrophages by co-culture with tumor cells. Thus, our indices reveal that tumor cell invasion is facilitated in a feed-forward manner by communication between tumor cells and macrophages in the tumor microenvironment.

## Introduction

Cellular migration and invasion are essential in many biological processes such as cancer metastasis^1–3^ and angiogenesis by endothelial cells^4^. Several assays have been developed to measure cellular invasion^3^; basically, the invasiveness of cells is measured by the distance or the path that cells travel in different setups such as Boyden chambers or wound healing assays. However, the cells are generally grown in two-dimensional (2D) *in vitro* space, which does not fully recapitulate the behavior of cells grown in three-dimensional (3D) *in vivo* tissue. Recently, spheroid-based 3D cultures have been widely employed in many applications, particularly drug discovery, as an *in vivo*-mimicking cellular platform^5–9^. In spheroid culture, cellular interactions among cells or with the extracellular matrix are better preserved^10,11^, and in addition, the physiological gradients inside of the tumor spheroid, such as oxygen, pH, and nutrients, create an environment that better mimics *in vivo*.

To date, most analyses of the invasiveness of cells grown in spheroids have been qualitative and descriptive rather than quantitative^12–14^. Raw data of cellular invasion usually takes the form of micrographs that are taken from bright field microscopy, with subsequent quantitative analysis of the micrographs relying on computational image processing accompanied by an impartial determination of the key parameters. Although there are good examples of image analysis of bright field micrographs, such as yeast growth^15^ and stem cell proliferation^16^ analyses, accurate quantitation based on bright field micrographs is challenging because of their low contrast. In particular, the edges of the invasive areas in cellular invasion assays are often not defined unambiguously. While recent quantitative analyses have employed diverse digital filters or computational parameters such as texture^4,17^, in many studies of cellular invasion, rather simple parameters such as the maximum distance that cells migrate or the area covered by the migrating cells are still preferred^18–22^.

Here, we provide novel quantitative indices for the invasiveness of cancer cells grown in spheroid culture. The indices are calculated from an image-based spheroid invasion assay that consists of (1) obtaining brightfield images with a standard inverted microscope, (2) setting objective threshold values for image analysis, and (3) segmenting a cell-covered area into a few discrete areas depending on cell density. First, we demonstrate our method with an invasive fibrosarcoma cell line HT1080, and compare our invasion indices to other conventional parameters. We then apply the method to investigate the cellular interactions between the lung carcinoma cell line LLC1 and the macrophage cell line Raw264.7 in different tumor microenvironments.

## Results and Discussion

### 3D culture and spheroid invasion assay

**Figure 1:**
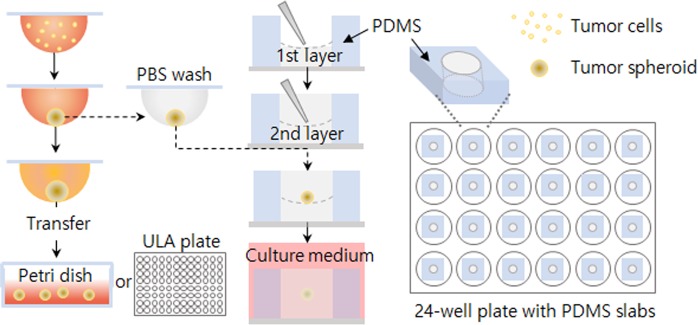
Spheroid formation and 3D tumor spheroid invasion assay. A schematic representation of spheroid formation and culture, and 3D invasion assay using a PDMS gasket attached to 24 well plate.

**Figure 2:**
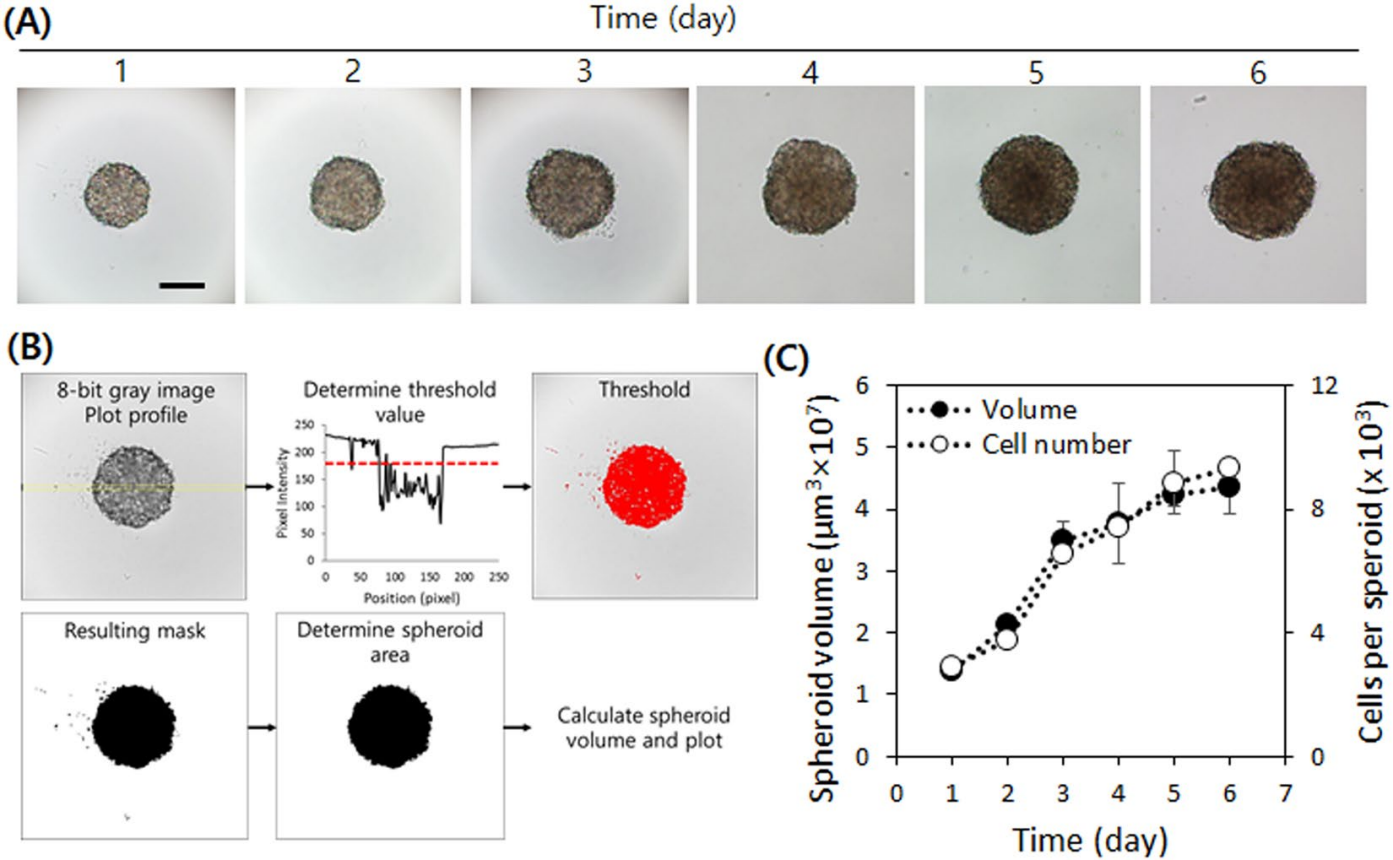
HT1080 spheroid growth and characterization. (**A**) A series of micrographs showing a spheroid growth in a hanging drop for days 1–3 and the low binding plate for days 4–6. (**B**) Image processing flow to calculate spheroid area and volume. The plot profile was obtained from the rectangle (yellow line) across the center of the spheroid and the threshold value (red dotted line) was set smaller than the background signal. After converting the image to binary, the area of the spheroid was obtained. (**C**) The cell numbers estimated by DNA content and the volume calculated by using (**B**) display a good correlation. The HT1080 spheroid grows rapidly for the first three days, then it continues growing with a reduced growth rate. Scale bar = 250 µm.

To measure the invasiveness of cells in 3D culture, we chose HT1080 fibrosarcoma cells, as they are highly invasive and frequently used to study cancer malignancy. For 3D culture, the hanging-drop method was employed, as described in Fig. 1. The HT1080 cells aggregated on the bottom of the drop due to gravity and formed a compact spheroid after 24 hours of culture (Fig. 2A and Supplemental Video 1). To characterize their growth, we estimated the volume of the spheroids from daily micrographs using the image-processing software ImageJ^23^. Volume was calculated from the area of a spheroid under the assumption of its spherical shape. For unbiased calculation of the area, we set a threshold based on a line profile of a greyscale image, as described in Fig. 2B. In brief, a bar was overlaid on the center of a spheroid to obtain the line profile. In a bright field micrograph, the object (spheroid) appears dark and the background is bright, and thus the pixel intensity of the background is consistently higher than that of the object. We thus set the threshold value to be slightly lower than the lowest background intensity to ensure comprehensive inclusion of the cell-covered area. Then we performed the command ‘Analyze Particles’ in ImageJ with the threshold set to minimum to exclude small particles (see the ImageJ macro for our image processing in Supplemental Information). We measured the number of cells in the spheroids from the DNA contents and found that the volume of the spheroids and the cell numbers showed similar trends (Fig. 2C). Both the volume and the cell number increased rapidly by day 3, and then slowed down, possibly due to limited oxygen and nutrients. Although spheroid growth was retarded after three days of culture, most of the cells appeared to be live.

**Figure 3:**
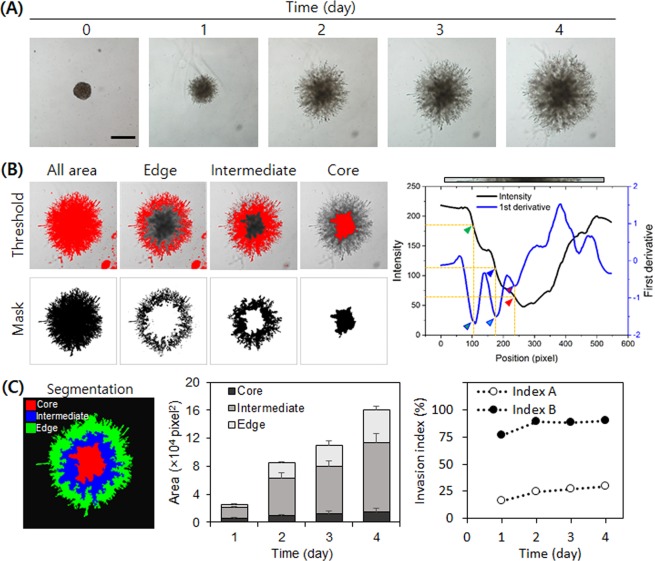
HT1080 spheroid invasion assay. (**A**) Daily micrographs showing the progress of invasion. (**B**–**F**) An example of analysis process using day 3 image. Scale bar = 500 µm. (**B**) Threshold settings (upper panel) for each segment and corresponding resulting binary images (lower panel). Threshold values were determined based on the intensity analysis (right panel). Green, blue, and red arrows with outer lines correspond to threshold values for all, intermediate, and core areas. (**C**) A segmented image of day 3 sample (left), time-course change of each area during the invasion assay (middle), and invasiveness indices (right).

Next, HT1080 spheroids were subjected to a 3D invasion assay under a laminin-rich condition. For the assay, we prepared an experimental setting using a polydimethylsiloxane (PDMS) gasket to minimize the volume requirement of Matrigel and to facilitate sample handling and imaging (Fig. 1). The small hole inside of the gasket requires less than 50 µL of Matrigel and the flat bottom of our 24-well plate provides good optical properties. The HT1080 cells started to invade the surrounding Matrigel in all directions within one hour (Fig. 3A, and Supplemental Video 2). To analyze the invasiveness, we divided the cell-covered area into three distinctive subareas: core, intermediate, and edge. The core is the darkest area in the center where cells aggregate tightly, the intermediate area is the grey zone where cells aggregate rather loosely, and the edge is where cells invade the vicinity in projections with no aggregation (Fig. 3B).

To achieve the successful segmentation of a spheroid into core, intermediate, and edge subareas, setting proper threshold values is a key step. Unlike well-contrasted spheroids in solution, invading cells in Matrigel exhibit lower contrast to the gel area due to the fewer number of cells. This difference in contrast hinders the setting of appropriate threshold values for each subarea in the image. To determine the region of interest objectively, we set the threshold values based on the first derivative of a pixel intensity plot that represents the degree of local intensity change. First, we draw an intensity profile from a long bar through the center of the spheroid. Then we obtain the first derivative profile after reducing local noise by smoothening the intensity profile by 20 pixels (blue line in the right panel of Fig. 3B). The size of the images in Fig. 3A is 580 by 580 square pixel, with 20 pixels corresponding to 64.5 µm, which includes approximately 6–7 cells.

We set three distinct threshold pixel intensities for all, intermediate, and core subareas using the first derivative profile of the pixel intensity plot. Peaks in the derivative profile inform big changes in pixel intensity. The left-most downward peak in Fig. 3B (green arrow) indicates the location of the boundary between the gel and the cell-covered area. We set the corresponding intensity (i.e. the 100 pixels, 180 intensity value position) as the threshold for the ‘all’ subarea. Then the next downward peak (blue arrow) appears at a position of 175 pixels and an intensity value of 120; this was set as the threshold for the ‘intermediate’ subarea. Lastly, the third downward peak (red arrow) appears at a position of 220 pixels and an intensity value of 70, which was set as the threshold for the ‘core’ area. Threshold values estimated from the right side of the graph were similar to those estimated from the left side of the graph; for consistency, we used the threshold values estimated from the left side of the graph throughout the study. The determined threshold values were applicable to all time-course images from the same set of experiments, and therefore batch processing using the ImageJ macro was achievable. In another set of experiments, the absolute threshold values changed slightly, but their ranges for each subarea remained around 10%. While the core area increased to a small extent, both the intermediate and the edge areas increased rapidly over time (Fig. 3C). In particular, the increase was dramatic between day 1 and 2.

Besides calculating each subarea to measure spheroid invasiveness, we defined invasion indices A and B (*I*_*inv*_*A* and *I*_*inv*_*B*) for comparison among samples in different conditions. Indices A and B are the percentage of edge subarea and the sum of edge and intermediate subareas over the entire cell-covered area, respectively.

$$\begin{array}{ccccc}Invasion\,index\,A({I}_{inv}A,\,{\rm{ \% }}) & = & \frac{{A}_{edge}}{{A}_{all}}\times 100 & = & \frac{{A}_{all}-({A}_{intermediate}+{A}_{core})}{{A}_{all}}\times 100\\ Invasion\,index\,B({I}_{inv}B,\,{\rm{ \% }}) & = & \frac{{A}_{edge}+{A}_{intermediate}}{{A}_{all}}\times 100 & = & \frac{{A}_{all}-{A}_{core}}{{A}_{all}}\times 100\end{array}$$

We found that both indices increased rapidly over the first two days of the spheroid invasion assay, but remained unchanged or only slightly increased over the following days. Since HT1080 cells are highly invasive, a large intermediate subarea was detected and consequently, index B was calculated to be much higher than index A (Fig. 3C).

### Comparison of invasion indices with parameters of conventional spheroid invasion assays

Although cellular invasion can be readily recognized by sight, a quantitative measurement is required for better comparison among samples in different conditions. Different analyses to quantify the invasiveness of tumor spheroids are typically performed, which can be categorized into three approaches. First, the most common analysis is to measure the cell-covered area, mainly a relative area to a control (i.e. day 0 or untreated sample)^4,18,19,22,24–26^. Second, measurements of the maximum length, or the distance from the edge of the initial spheroid to the outermost edge of the protruding cells, are often performed^21,27,28^. Lastly, the number of sprouts or detached cells from the spheroid body has also been employed as a parameter for invasiveness^4,24,28^. In terms of the first approach, while the total cell-covered area is a reasonable parameter to measure invasiveness, often the selection of the ‘invasive cell’ area tends to be subjective. In addition, increases in spheroid area by proliferation and by invasion cannot be discriminated. Measuring the maximum length of the invasion area as in the second approach is a simple and quick method, but it has the same limitation as the area calculation because it only measures the distance from the center to the tip of the cell-covered area without considering cell proliferation. Furthermore, these methods are not appropriate to measure cellular invasiveness of non-spherical aggregates. Regarding the third approach, while the number or length of sprouts provides a reasonably good indication of invasiveness, it often is insufficient because this parameter solely reflects the complexity of the edge.

**Figure 4:**
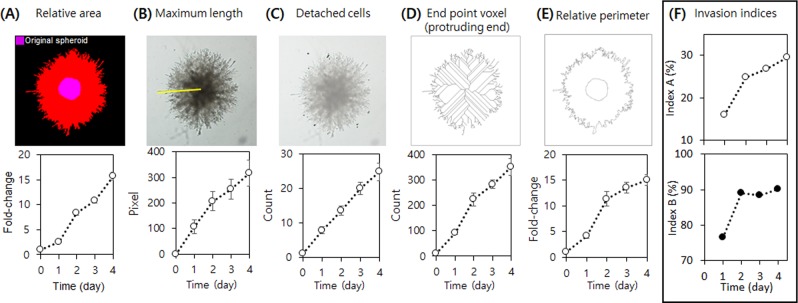
Comparison of time-course plots of conventional spheroid invasion parameters (**A**–**E**) and our invasion indices (**F**) for HT1080 spheroid invasion assay.

We compare the aforementioned conventional invasion parameters with our invasion indices using the same set of images in Fig. 3A. The relative area, maximum length, and the number of detached cells parameters tend to steadily increase during the invasion assay but fail to capture the initial invasion from the edge of the spheroids during the first 24 hours (Fig. 4A–C). On the other hand, the number of end-point voxels, which corresponds to the number of sprouts, showed a reduced increase after day 2 (Fig. 4D). Unlike the others, this parameter reflects changes in the interface between the cells and matrix (i.e. Matrigel). We plotted another parameter to reflect these interfacial changes—relative perimeter. While the number of end-point voxels only reflects changes in the invasive front, the relative perimeter reflects both the expansion of the cell-covered area and the geometric complexity resulting from the protruding (or invading) cells. Interestingly, the relative perimeter displayed a similar trend to invasion index A, indicating that this parameter can provide for a quick and easy analysis of spheroid invasion.

The most striking difference between our indices and the conventional parameters is that our analysis showed that invasion begins at day 1 and that the indices do not change much over the course of the assay, whereas the other parameters continuously increase over time. Conventional parameters cannot provide detailed information on spheroid invasion, such as core area expansion by cell proliferation that is frequently observed in less invasive cells. Thus, we conclude that our indices can present the cells’ invasion capacity at the time of measurement, in contrast to the conventional parameters as cumulative indices of all invasion events confounded by proliferation events. We note though that the parallel analysis of multiple parameters can provide better insights into cellular behaviors. For example, Guiet *et al*. analyzed spheroid areas, the number of detached cells from spheroids, and the maximal invasion distance reached by cells to study the impact of macrophages on the invasion of tumor cells^24^.

### Facilitation of 3D invasion by tumor–macrophage communication

We then applied our method to quantify the invasiveness of cancer cells grown together with macrophages, which is important to form a tumor microenvironment^29^. The reciprocal communication between cancer cells and macrophages has been well documented; in this study, we focus on interactions between lung cancer cells and macrophages^30–32^ and pursue a quantitative analysis of the 3D invasion of a cancer spheroid. A mouse Lewis lung carcinoma LLC1 cell line was co-cultured in spheroids with Raw264.7, a mouse macrophage cell line, or treated with Raw264.7 cell-conditioned medium.

**Figure 5:**
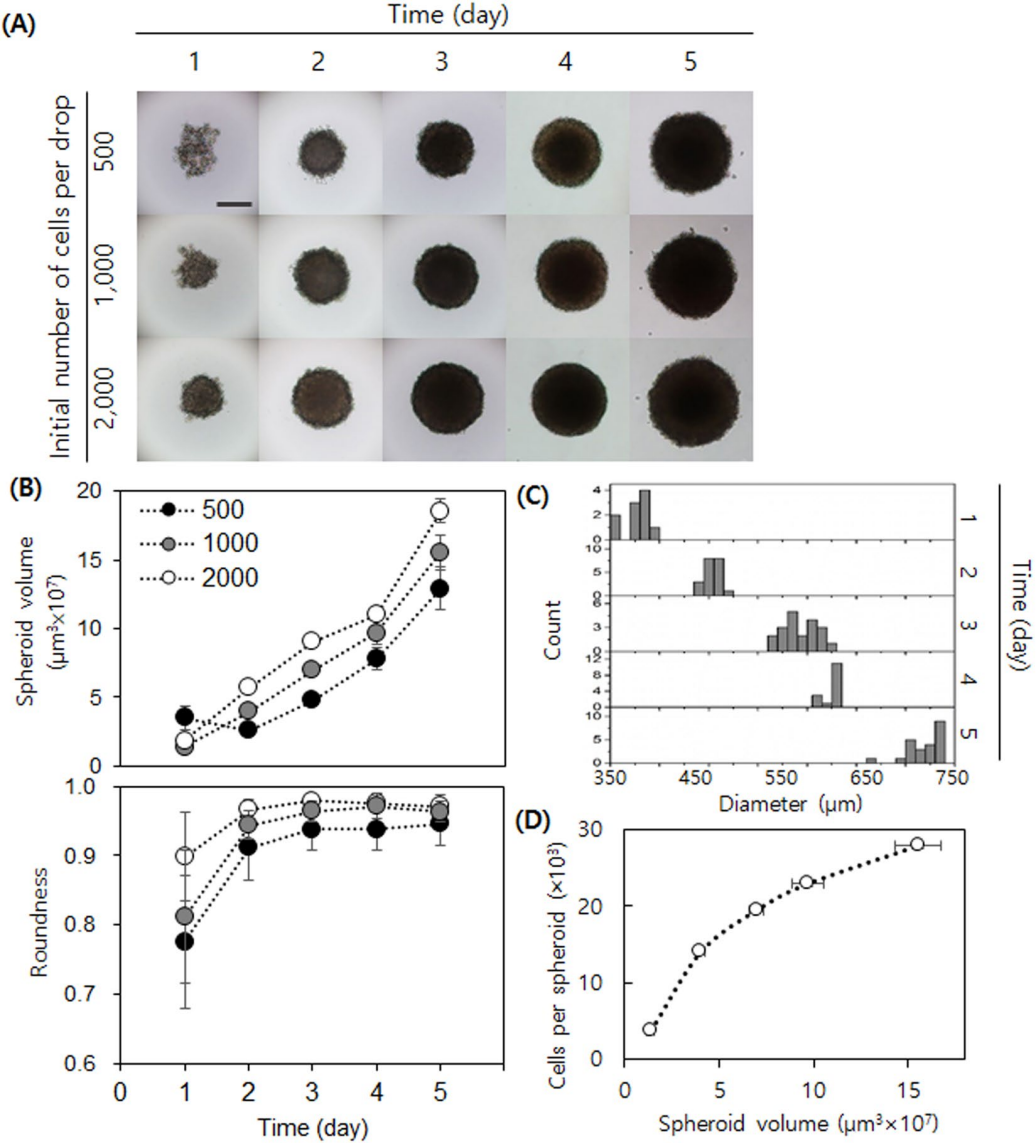
LLC1 spheroid growth and characterization. (**A**) Daily micrographs showing the spheroid growth in a hanging drop (for the first three days) and low binding plate (latter two days). Scale bar = 250 µm. (**B**) Daily plots for volume and roundness of spheroid. (**C**) Daily histograms of spheroid diameter (initial cell number = 1,000) shows that the size of spheroids remains uniform throughout the culture. (**D**) The plot between cell number and the calculated volume.

First, we tested the effect of different initial cell numbers and the addition of methylcellulose on spheroid formation and growth in a hanging drop. An array of drops containing 500, 1,000, and 2,000 cells per 25 μL culture medium was prepared and monitored daily for five days. The addition of methylcellulose was required to rapidly form a single spheroid (Fig. S1, Supplemental Information). Also, the larger the starting cell number, the faster the spheroid was formed (Fig. 5A), with most of the cells in a given spheroid at day 2 were viable (Fig. S2, Supplemental Information). The LLC1 spheroids grew continuously, forming dense cores, and became more spherical over time (Fig. 5B). The histograms of spheroid diameter displayed a uniform distribution in size over the culture period. We choose 1,000 cells as the initial cell number because the cells in this sized drop formed a compact and uniform spheroid with high viability by day 2. Also, the roundness of the spheroids with 1,000 initial cells was comparable to that of the spheroids with 2,000 initial cells, and the size distribution of the spheroids was stably maintained with only small deviation throughout the culture period (Fig. 5C). We performed live imaging of LLC1 spheroid formation and growth (Supplemental Video 3), in which the video clearly shows the initial spheroid aggregation and growth. As inferred from the non-linear correlation between spheroid volume and number of cells per spheroid as estimated from DNA content (Fig. 5D), the shape of spheroids may not be perfectly spherical.

**Figure 6:**
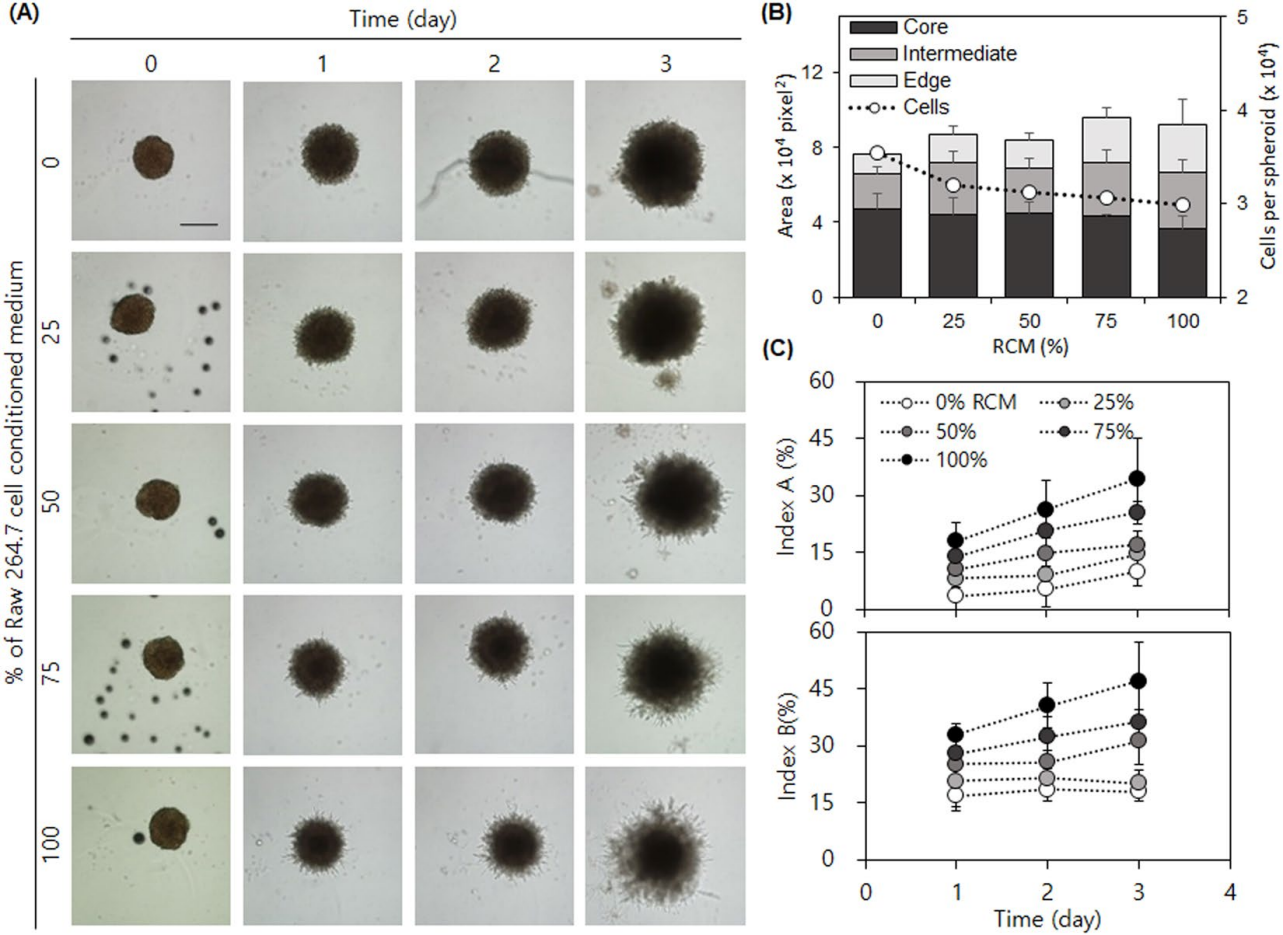
LLC1 spheroid invasion assay. (**A**) Daily micrographs of spheroid invasion under different concentrations of RCM. (**B**) The analyzed area for each segment and cell number estimated by DNA content at day 3. The number of cells showed a good correlation to the core area. (**C**) Time-course changes of invasiveness indices. Scale bar = 200 µm.

Then, we investigated changes in the invasiveness of an LLC1 spheroid embedded in Matrigel in the absence and presence of Raw264.7 cells. We decided to use a medium conditioned with Raw264.7 rather than add macrophages directly to cell cultures because co-cultured macrophages hindered the proper imaging of LLC1 invasion. Embedded LLC1 spheroids in a normal culture medium (control) continued to grow and exhibited minimal invasion at the edge of the spheroid (short protrusion) even after day 3. On the other hand, when the spheroids were cultured with 100% Raw264.7 cell-conditioned medium (RCM), protruding cells appeared much more quickly, even at day 1 (Fig. 6A). Time-course videos clearly show the difference in cellular invasion between the LLC1 cells in control and RCM (Supplemental Videos 4 and 5 and Fig. S3). In control, only a few cells detached from the edge of the spheroid body and moved around to a minor extent, but later merged back to the spheroid body where cells were densely packed. In the RCM environment though, a larger number of cells detached from the edge and actively moved within channels in the Matrigel (Supplemental Videos 6 and 7). To confirm that the observed cellular invasion was induced by paracrine signaling through the RCM, we performed our developed spheroid invasion assay with different concentrations of RCM. As RCM content increased, the level of invasion increased, and the size of the dark core decreased.

We analyzed daily micrographs by calculating our aforementioned invasion indices. The intermediate and edge subareas of the LLC1 spheroids increased as RCM content increased, indicating that the soluble components secreted by Raw264.7 cells facilitated invasion. Interestingly, the core subarea decreased as the RCM content increased. When RCM content was low, spheroids showed larger core subareas suggesting higher levels of cell proliferation. These results led us to ask whether RCM content increases invasion at the expense of cell proliferation. Indeed, as the RCM content increased, the total number of cells (open circles in Fig. 6B) decreased while the total cell-covered area (overall bar height in Fig. 6B) increased, supporting our hypothesis that invasion and proliferation are inversely related^33–35^. The invasion indices A and B were small compared to those of the HT1080 cells, indicating that LLC1 cells are less invasive (Fig. 6C). While *I*_*inv*_*A* of HT1080 cells increased rapidly until day 2 and then remained unchanged, *I*_*inv*_*A* of LLC1 cells steadily increased in both RCM content and in a time-dependent manner. Although *I*_*inv*_*B* remained unchanged without addition of RCM over the culture period, *I*_*inv*_*A* increased over time, differing from HT1080 cells. Interestingly, RCM addition enhanced both indices in terms of RCM content and time-dependence, providing validation that our indices are faithful parameters for examining invasion processes as well as the role of macrophages in tumor microenvironments. Other parameters for spheroid invasion assays—relative area and relative perimeter—were calculated and compared with our indices. The relative area did not significantly change along with the change in RCM content, while the relative perimeter changed in an RCM dose-dependent manner (Fig. S4, Supplemental Information). Again, the trend in the relative perimeter was similar to that of *I*_*inv*_*A* as observed in the HT1080 spheroid invasion assay, indicating that relative perimeter, but not relative area, can serve as a reasonable parameter for cellular invasion measurement.

**Figure 7:**
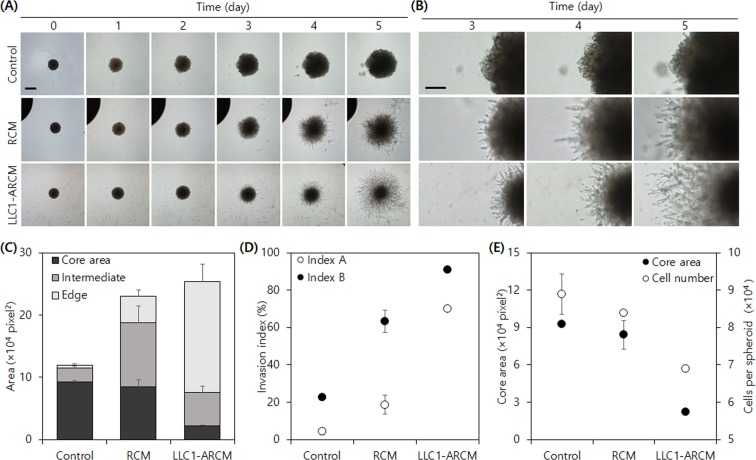
LLC1 spheroid invasion assay using activated Raw264.7 cell conditioned medium. (**A**) Daily micrographs of spheroid invasion of LLC1 under different conditioned media. Scale bar = 500 µm. (**B**) Enlarged view on the edge of spheroids. Scale bar = 250 µm. (**C**) Subareas, (**D**) invasion indices, and (**E**) co-plot of core area and cell number per spheroid at day 5.

Lastly, we investigated the effect of a conditioned medium from activated Raw264.7 cells on the invasiveness of LLC1 spheroids. It has been reported that macrophages are activated by tumor cells and assist tumor progression in tumor microenvironments^29^. Raw264.7 cells displayed activated morphology when cultured with tumor-conditioned media prepared with four different types of cancer cell lines: LLC1, a mouse pancreatic cancer cell line Panc02, a human breast cancer cell line MDA-MB-231, and HT1080 (Fig. S5, Supplemental Information). Interestingly, conditioned media prepared from activated Raw264.7 cells (ARCM) by LLC1 strongly promoted the invasion of embedded LLC1 spheroids even compared with the cells treated in 100% RCM, suggesting that tumors can facilitate their invasion capability in a feed-forward manner by enhancing the pro-invasion effect of Raw264.7 cells by their activation through tumor-secreted molecules (Fig. 7).

The LLC1-ARCM treated spheroids showed much smaller cores, indicating decreased cell proliferation. Indeed, the total number of cells in LLC1 spheroids in LLC1-ARCM was also significantly smaller than those in control and RCM conditions. These findings further support our hypothesis that tumor cell proliferation is subdued during active invasion. While all the tested ARCMs induced a high level of invasion in LLC1 spheroids, LLC1- and Panc02-ARCMs were more effective than other ARCMs in promoting LLC1 spheroid invasion (Fig. S6, Supplemental Information). Remarkably, the core subareas of LLC1 spheroids in all tested ARCMs were much smaller than those in control and RCM conditions. Although the total cell-covered areas in MDA-MB-231–ARCM and HT1080-ARCM were approximately 80% of those in RCM, the invasion indices were much higher because of the difference in the extent of cell proliferation between the conditions. Additional comparison of our indices with total area, the most commonly employed spheroid invasion parameter, demonstrated that the change in total area failed to distinguish area expansion by cell proliferation or invasion, whereas our indices distinguished the difference. Also, the trend in the relative perimeter was similar to that of *I*_*inv*_*A* as observed in the other experiments (Fig. S7, Supplemental Information). Although relative perimeter has not been commonly employed in spheroid invasion assays, assuming that micrographs are well-focused and image analysis is performed properly, relative perimeter may serve as a better invasion parameter than relative area because it better reflects the complexity of the invading edge, as we observed in our study. However, more informative parameters are preferred to understand spheroid invasiveness; the segmentation-based approach facilitates the deciphering of other cellular processes in detail, such as angiogenesis, as described in the work by Blacher *et al*.^4^. The adoption of computer-based segmentation could therefore further assist the automation of quantification^14^.

In sum, we believe that our invasion indices could serve as reliable and precise parameters to specifically quantify the invasiveness of tumor cells in *in vitro* tumor microenvironments. We are in active investigation of the mechanism by which proliferation and invasion are counter-regulated, and of the secretomes of tumor cells that power up the activity of macrophages to drive tumor invasion. Considering recent reports on cell deformability and proliferation^36^ as well as cell cycle and invasive behavior^37^, investigation of cell cycle duration in an invasion assay could provide valuable insight into the inverse relation between proliferation and invasion.

## Methods

### 2D monolayer and 3D spheroid culture

Human fibrosarcoma cell line HT1080, mouse lung adenocarcinoma cell line LLC1, mouse macrophage cell line Raw264.7, and human breast cancer cell line MDA-MB-231 were obtained from ATCC. Panc02 was a kind gift from Prof. Christiane J. Bruns (University of Cologne, Germany). The cells were maintained in DMEM supplemented with 10% FBS, 200 units/mL penicillin, 200 units/mL streptomycin, 2 mM Glutamax, and 10 mM HEPES (Hyclone) at 37 °C in a humidified 5% CO_2_ atmosphere. All culture supplies were obtained from Thermo Fisher Scientific, unless otherwise indicated.

Dissociated cells from monolayer culture were suspended in a culture medium containing 0.24% (w/v) methylcellulose (Sigma) at desired densities ranging from 500 to 2,000 cells per 25 μL. The drops were placed on the lid of a 150 mm culture dish using a multichannel pipette. The lid was then inverted and placed on the bottom of the dish filled with 20 mL of 1x PBS. After four days in the drop, spheroids were transferred to a petri dish.

Spheroid images were obtained using an inverted microscope (Olympus IX71) by focusing on where the edge cells were clearly visible.

### Proliferation assay by DNA content

Cells from 2D or 3D spheroid culture were harvested daily and the pellets were kept at −70 °C overnight. Respectively ten and three spheroids were collected in hanging-drop and Matrigel culture. In the case of the Matrigel-embedded spheroids, we removed the Matrigel before harvesting the cells, as follows. In brief, we removed the cell culture media and stored the plate at −20 °C for an hour to harden the Matrigel block in the PDMS slab. Then the Matrigel block was removed from the slab and subjected to Cell Recovery Solution (Corning) at 4 °C overnight to liquify the Matrigel. The next day, the number of cells were estimated using a CyQUANT^®^ cell proliferation assay kit (Life Technologies) according to manufacturer instructions. In brief, a standard curve from a series of known cell numbers was prepared.

### Spheroid viability assay

For a viability assay by imaging, LLC1 spheroids were washed three times with 1x PBS and incubated with 2 μM Calcein-AM and 0.5 μM ethidium homodimer in 1x PBS for 10 min in the dark. Images were captured using an inverted microscope. For a viability assay via trypan blue exclusion, 20 LLC1 spheroids were collected, washed with 1x PBS, and incubated with Accutase for 5 min. Then we further dissociated the cell clumps by pipetting about 40 times using a low-binding tip to prevent cell loss. Viability was obtained by a cell counter (Countess).

### Time-lapse imaging

We used a compact imaging device (Juli Br, NanoEnTek), which can be accommodated in a conventional CO_2_ incubator, to record the formation, growth, and invasion of LLC1 spheroids. Time-series images were obtained at intervals of 30 min for 72 h.

### Preparation of conditioned media and activation of Raw264.7

Raw264.7 cells were seeded in a 6-well plate (1 × 10^5^ cells per well). After 48 h, we harvested the conditioned medium and removed cell debris by centrifugation. The conditioned medium was then sterilized by filtration (0.22 μm syringe filter) and stored at −80 °C up to one week before use. In the case of cancer cells, cells were seeded in a 150 mm dish (2 × 10^6^ cells per dish) with the conditioned media prepared in the same manner as for the Raw264.7 cells. To obtain conditioned media from activated Raw264.7 cells, we treated Raw264.7 cells grown for 24 h in a 6-well plate (1 × 10^5^ cells/well seeding density) with various cancer cell conditioned media. After 24 h, the conditioned media were prepared in the same manner as for the Raw264.7 cells.

### 3D spheroid invasion assay

All materials for the assay (Matrigel, phenol red-free (Corning), micropipette tips, 24-well plate, and PDMS well) were pre-chilled overnight. We made holes 5 mm in diameter on the PDMS slab (2.5 mm in height) using a biopsy punch. Twenty-five microliters of Matrigel was cast in the PDMS holes and solidified for 40 min in an incubator. Then we added 25 μL of Matrigel on top of the first layer of Matrigel and carefully placed a PBS-washed spheroid in the center. After solidifying the gel in an incubator for 30 min, the well plate containing the PDMS slabs was filled with a culture media. We took micrographs at intervals of 24 h without changing the culture medium.

### Image analysis

All image analysis was performed by ImageJ (NIH, v1.48) except for detached cell counting, which was analyzed by Fiji, an open-source image processing package based on ImageJ. Examples of macro scripts for batch processing can be found in Supplemental Information. Briefly, images were converted to 8-bit greyscale and cropped to desired sizes. The cropped images were then converted to binary images with appropriate threshold values. Parameters for the binary mask, such as area and perimeter, were analyzed using the ‘Analyze Particles’ function. For end-point voxel calculation and detached cell counting, the ‘Skeletonize’ function of ImageJ and ‘3D object count’ function of Fiji were used, respectively.

### Statistical analysis

Experiments were independently repeated at least in triplicate. Error bars in the graphical data represent standard deviations.

## Supplementary information

Supplemental Information.

Supplemental video 1.

Supplemental video 2.

Supplemental video 3.

Supplemental video 4.

Supplemental video 5.

Supplemental video 6.

Supplemental video 7.

## Data Availability

The datasets generated during the current study are available from the corresponding author on reasonable request.
