# Supplementary material for: Novel invasion indices quantify the feed-forward facilitation of tumor invasion by macrophages
Source: Sci Rep. 2020 Jan 20;10:718. doi: 10.1038/s41598-020-57517-6 (PMC6971071; doi:10.1038/s41598-020-57517-6)
Supplement: Supplementary file 1 — Supplemental Information. [file 41598_2020_57517_MOESM1_ESM.pdf]

Supplemental Information

Novel invasion indices quantify the feed-forward facilitation of tumor invasion by macrophages

Gippeum J. Lim[1], Suk-Jo Kang[1*], and Ji Youn Lee[2*]

[1]Department of Biological Sciences, Korea Advanced Institute of Science and Technology, 291 Daehak-ro, Yuseong-gu, Daejeon 34141, Republic of Korea.

[2] Center for Bioanalysis, Division of Chemical and Medical Metrology, Korea Research Institute of Standards and Science, 267 Gajeong-ro, Yuseong-gu, Daejeon 34113, Republic of Korea

*Corresponding authors (JYL, SJK)

Phone: +82-42-868-5363 (JYL); +82-42-350-2611 (SJK)

Fax: +82-42-868-5801 (JYL); +82-42-350-2610 (SJK)

E-mail: jylee@kriss.re.kr; suk-jo.kang@kaist.ac.kr

Lists

1. Supplemental figures, Figs. S1–S7
2. List of supplemental videos
3. Scripts for ImageJ macros

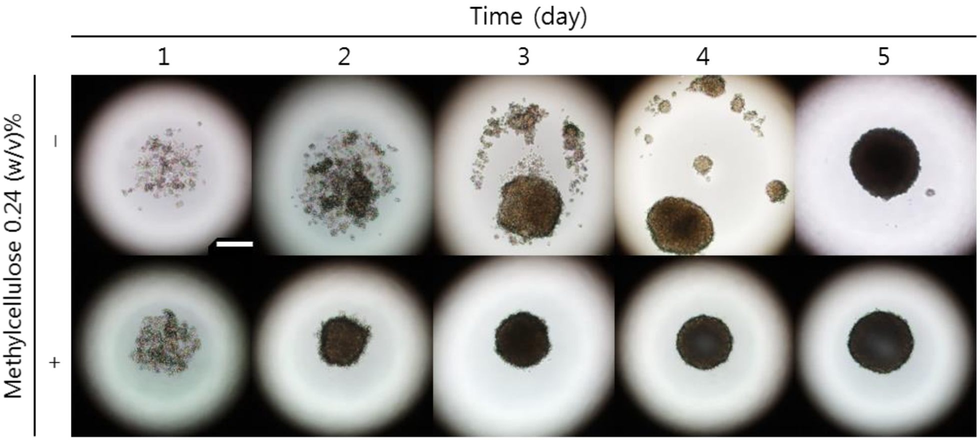

Figure S1. Effect of methylcellulose on LLC1 spheroid formation. In the presence of methylcellulose, LLC1 cells formed spheroids more quickly. The initial number of cells was 500 cells per 25 µL drop. Scale bar = 500 µm.

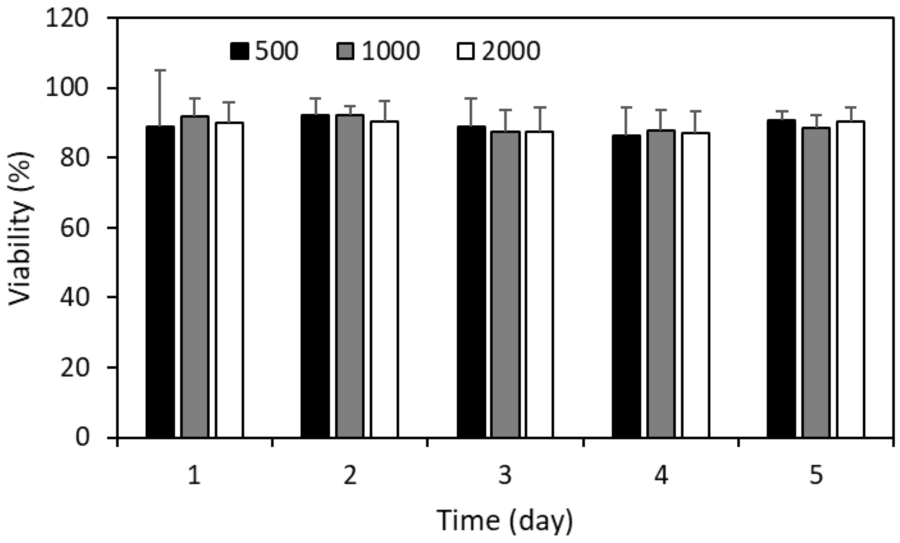

Figure S2. LLC1 spheroid viability. Regardless of the initial cell number, the cell viability was maintained over 85% during the culture up to 5 days.

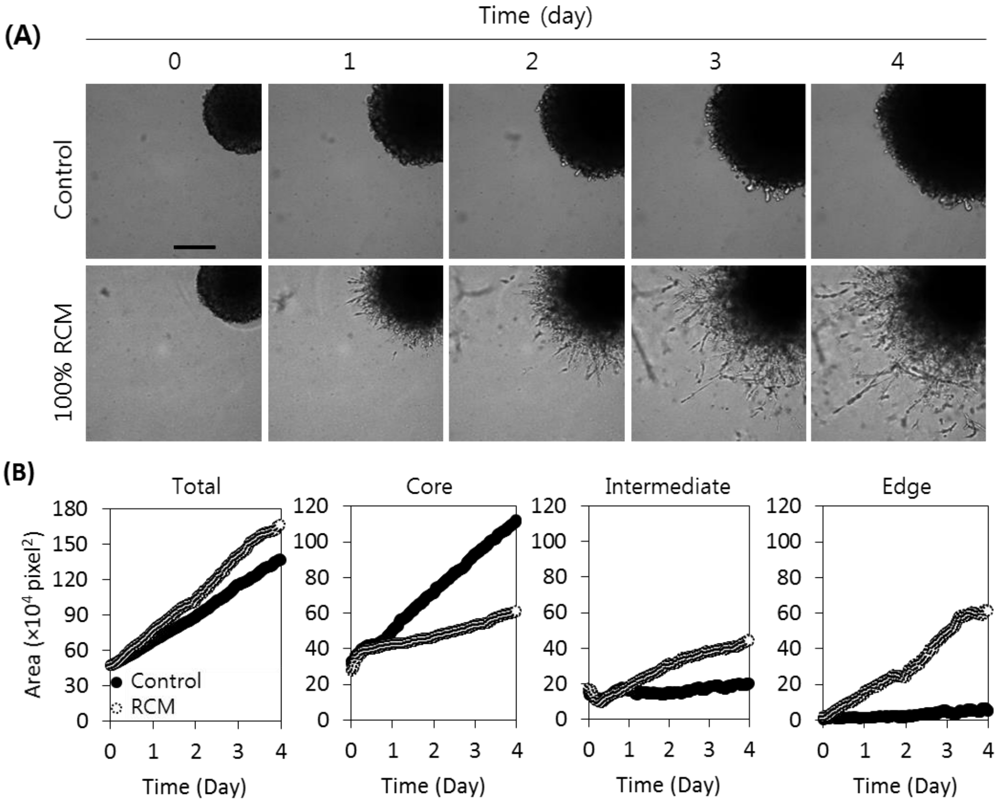

Figure S3. Continuous monitoring of subareas using a live cell analyzer. (A) Representitiave images of a spheroid in different media for days 0–4 and (B) corresponding plots for total and subareas. In the control, only the core subarea continuously increased over time while the intermediate and edge subareas unchanged. On the other hand, in RCM conditionScale bar = 250 μm.

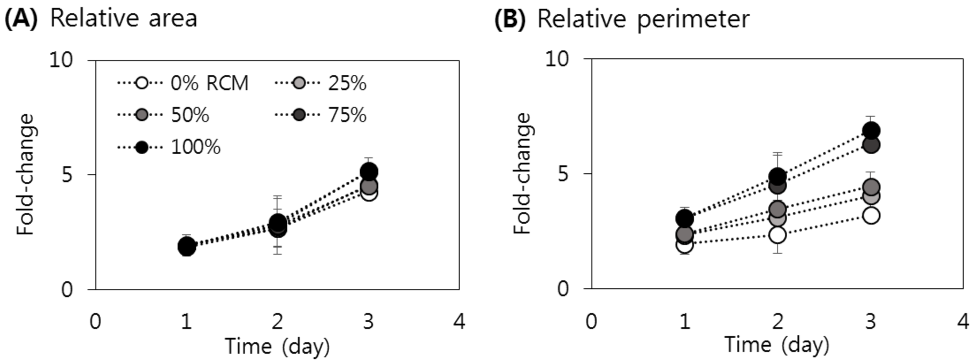

Figure S4. (A) Relative area and (B) relative perimeter fold-changes in the LLC1 spheroid invasion assay with different contents of Raw264.7 cell conditioned medium.

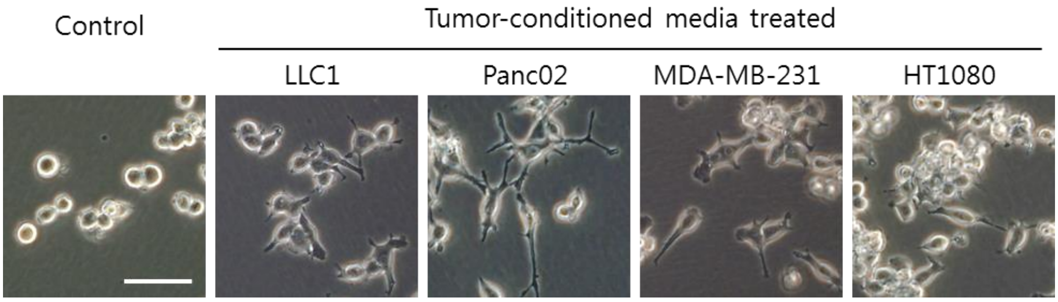

Figure S5. Morphological changes of Raw 264.7 cells by various cancer-conditioned media at 24 h. Micrographs of Raw264.7 cells grown in a normal cell culture medium (control) and in the indicated tumor cell-conditioned media are shown. Scale bar = 50 μm.

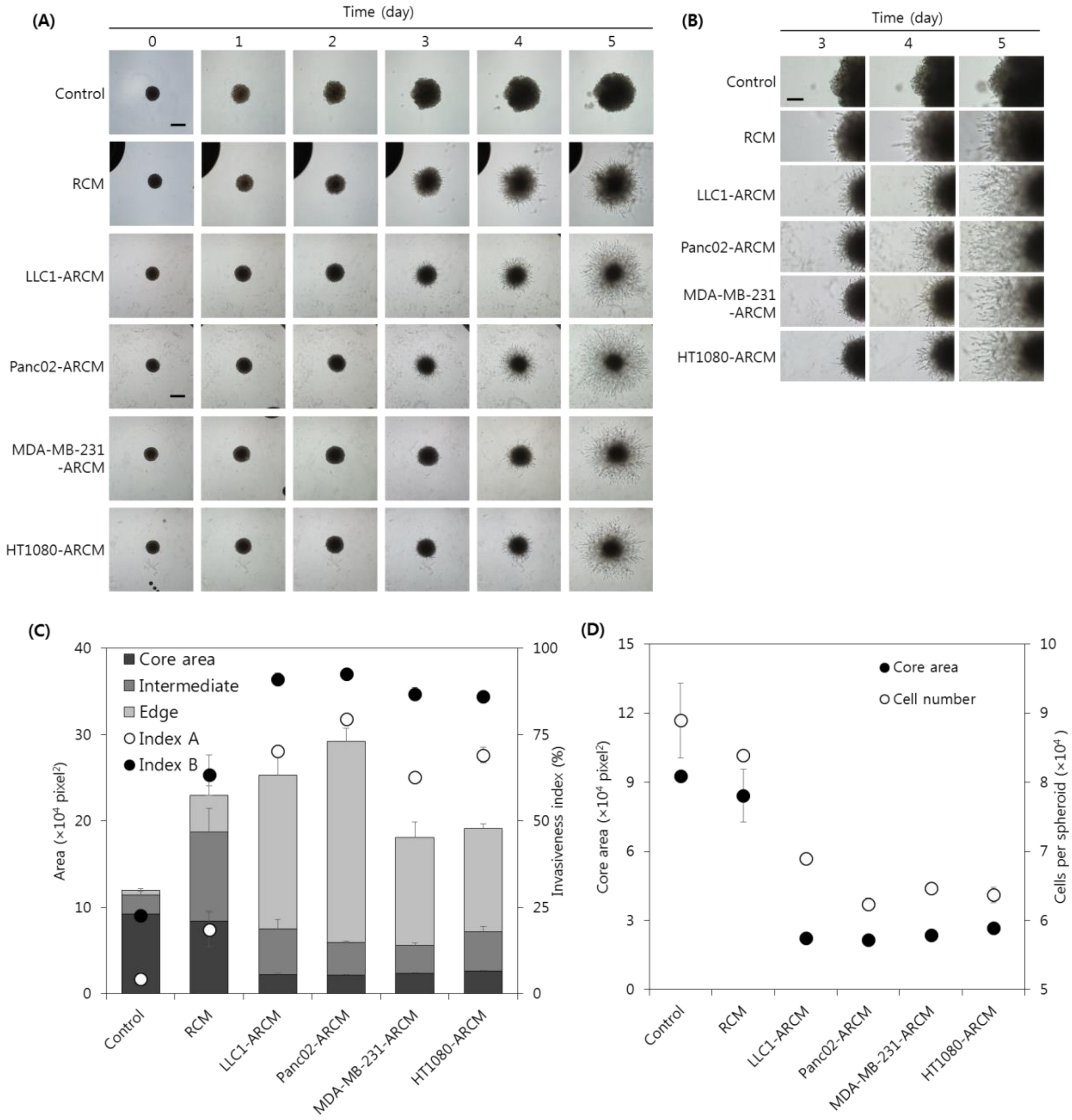

Figure S6. LLC1 spheroid invasion assay using activated Raw264.7 cell conditioned media. (A) Daily micrographs of spheroid invasion under different conditioned media. Scale bar = 500 μm. (B) Zoomed images of the edge subareas at days 3–5. Scale bar = 250 μm. (C) Subarea and invasiveness indices at day 5. (D) Co-plot of core subarea and cell number at day 5.

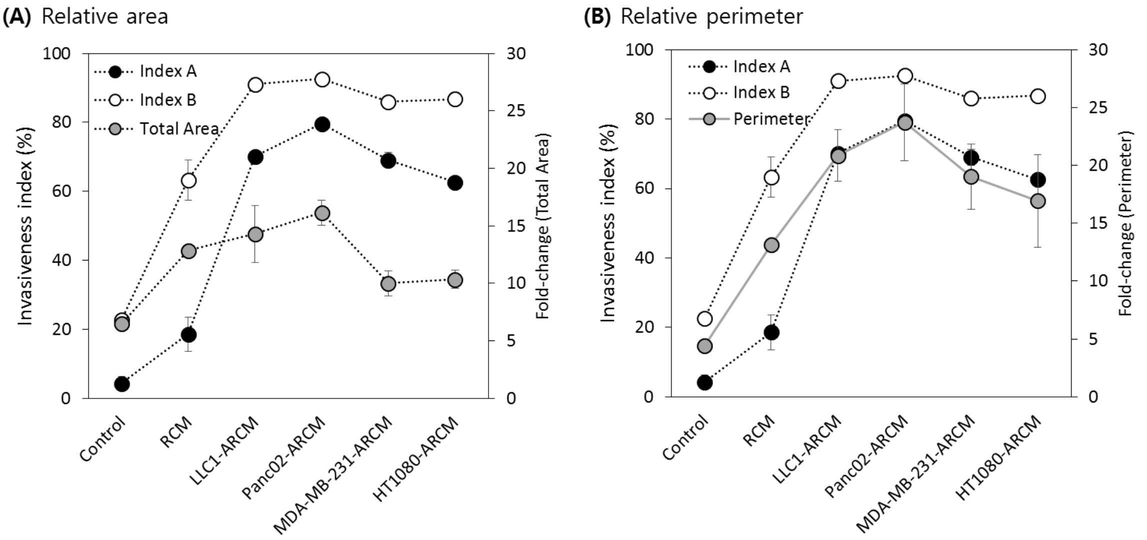

Figure S7. (A) Relative area and (B) relative perimeter fold-changes in the LLC1 spheroid invasion assay in different RCM and ARCM conditions. In tested conditions, while the relative area shows a different pattern compared to our invasion indices, relative perimeter appears similar to our indices.

List of supplemental videos

Supplemental video 1: HT1080 spheroid growth in a hanging drop

Supplemental video 2: HT1080 spheroid invasion in Matrigel

Supplemental video 3: LLC1 spheroid growth in a hanging drop

Supplemental video 4: LLC1 spheroid invasion in complete medium

Supplemental video 5: LLC1 spheroid invasion in RCM

Supplemental video 6 and 7: LLC1 spheroid invasion in RCM highlighting an edge subarea showing dynamic invasion process

Examples of ImageJ macro script

1. HT1080 area calculation

```
makeRectangle(x, y, 300, 300);   // select area of interest, 300 square pixels (x, y, width, height)
run("Crop");                     // crop the area of interest
run("8-bit");                    // convert to a grey image
setThreshold(0, t);              // set threshold value (t, e.g. 180) to make a binary image
setOption("BlackBackground", false);
run("Convert to Mask");          // convert to a binary image
run("Analyze Particles...", "size=1000-Infinity show=Masks exclude");
                                 // size exclusion smaller than 1,000 square pixel
run("Fill Holes");               // fill holes in spheroid area (if any)
run("Analyze Particles...", "size=1000-Infinity show=Outlines display exclude");
                                 //obtain area information
```

2. Segmentation

```
makeRectangle(x, y, 580, 580);   // select area of interest, 580 square pixels (x, y, width, height)
run("Crop");                     // crop the area of interest
run("8-bit");                    // convert to a grey image
run("Threshold...");             // set threshold value to make a binary image
setThreshold(0, value);          // values for core: 20–55; extended core: 70–125; all: 100–185
setOption("BlackBackground", false);
run("Convert to Mask");          // convert to a binary image
run("Analyze Particles...", "size=1000-Infinity show=Masks exclude");
                                 // size exclusion smaller than 1,000 square pixel
run("Fill Holes");               // fill holes in spheroid area (if any)
run("Analyze Particles...", "size=1000-Infinity show=Nothing display exclude");
                                 //obtain mask images and area information
```

3. Skeletonize (to obtain end-point voxels)

```
makeRectangle(x, y, 750, 750);   // select area of interest, 580 square pixels (x, y, width, height)
run("Crop");                     // crop the area of interest
run("8-bit");                    // convert to a grey image
```

```
setThreshold(0, t);                    // set threshold value (t, e.g. 180) to make a binary image
setOption("BlackBackground", false);
run("Convert to Mask");                // convert to a binary image
run("Analyze Particles...", "size=1000-Infinity show=Masks exclude");
                                       // size exclusion smaller than 1,000 square pixel
run("Fill Holes");                     // fill holes in spheroid area (if any)
setOption("BlackBackground", false);
run("Skeletonize");
run("Analyze Skeleton (2D/3D)", "prune=none calculate display");
selectWindow("Longest shortest paths");
selectWindow("Mask-labeled-skeletons");
                                       // obtain a skeletonized image and end-point voxel information
```
